# Circular RNA circ_0008365 regulates SOX9 by targeting miR-338-3p to inhibit IL-1β-induced chondrocyte apoptosis and extracellular matrix degradation

**DOI:** 10.1186/s13018-022-03240-z

**Published:** 2022-10-14

**Authors:** Shengbin Shuai, Qianqian Cai, Yunxia Ou

**Affiliations:** 1 Department of Rehabilitation, Tianjin Hospital, Tianjin, 300211 China; 2 Pain Department, Chongqing Qijiang District People’s Hospital, No. 54, Tuowan Branch Road, Gunan Street, Qijiang District, Chongqing, 401420 China

**Keywords:** circ_0008365, miR-338-3p, SOX9, Osteoarthritis

## Abstract

**Background:**

Osteoarthritis (OA) is a chronic disease that involves chondrocyte injury and dysfunction. CircRNAs participate in OA progression, but the roles of circRNAs in the occurrence of OA are unclear. In this study, we explore the role of circ_0008365 in OA.

**Methods:**

CHON-001 cells were treated with interleukin-1β (IL-1β) to construct an in vitro OA cell model. The levels of circ_0008365, SRY-related high mobility group-box gene9 (SOX9) mRNA, and microRNA-338-3p (miR-338-3p) were detected by quantitative real-time polymerase chain reaction (qRT-PCR) assay. Western blot (WB) assay was used to measure protein levels. Cell Counting Kit-8 (CCK-8) assay, 5-ethynyl-2′-deoxyuridine (EDU) assay, and flow cytometry analysis were used to detect cell viability, proliferation, and apoptosis, respectively. Dual-luciferase reporter assay, RNA pull-down assay, and RNA immunoprecipitation (RIP) assays were used to confirm the interaction between miR-338-3p with circ_0008365 or SOX9.

**Results:**

Circ_0008365 expression was reduced in OA tissues and IL-1β-induced CHON-001 cells. Functionally, circ_0008365 inhibited viability, proliferation, and ECM degradation and promoted apoptosis of IL-1β-induced CHON-001 cells. Mechanistically, circ_0008365 acted as a sponge of miR-338-3p to regulate SOX9 expression, thus exerting its functions in IL-1β-induced CHON-001 cells. Moreover, exosomal circ_0008365 had great value in diagnosing OA.

**Conclusion:**

Circ_0008365 alleviates IL-1β-induced CHON-001 cell damage through the miR-338-3p/SOX9 axis, which suggested that circ_0008365 might be a new therapeutic target for OA.

**Supplementary Information:**

The online version contains supplementary material available at 10.1186/s13018-022-03240-z.

## Introduction

Osteoarthritis (OA) is a common osteoarticular disease with the main characteristics including synovial inflammation, cartilage degeneration and destruction, and cartilage bone remodeling. About 15 million new cases were diagnosed with OA in 2017 around the world [1]. Complicated clinical symptoms and difficult treatment bring a heavy burden to patients and society [2]. However, there is currently no effective treatment for OA. Thus, it is urgent to develop a new therapeutic strategy for OA.

Circular RNAs (circRNAs) are a kind of non-coding RNAs with stable closed-loop structures [3], which are abnormally expressed in a variety of diseases [4–6]. Increasing research reports showed that circRNAs were involved in OA progression [7–9]. For instance, circRNA FADS2 was lowly expressed in OA and inhibited apoptosis of LPS-treated chondrocytes [10]. CircRUNX2 was found in the serum of OA patients and identified with diagnostic value in OA [11]. CircCDH13 was up-regulated in OA cartilage tissues and could promote OA progression by regulating the miRNA-296-3p/PTEN pathway [12]. Circ_0136474 contributed to OA progression by modulating miR-127-5p and MMP13 [13]. CircRNA circ_0005105 accelerated the ECM degradation of IL-1β-treated chondrocytes by sponging miR-26a and up-regulating NAMPT [14]. Moreover, a previous report showed that circ_0008365 (Position: chr2: 224856519-224866639, Length: 707 nucleotides, Host gene Symbol: SERPINE2) was down-regulated in an OA model [15]. However, the function of circ_0008365 in OA occurrence remains unclear.

In addition to circRNA, other non-coding RNAs, such as microRNA (miRNA) and small interfering RNA (siRNA) are also involved in the occurrence of osteoarticular diseases [16–19]. MiRNA is a small non-coding RNA that mediates mRNA transcription and degradation by competitively binding to the 3′-UTR of mRNAs [20, 21]. CircRNAs could participate in OA progression by sponging miRNAs, thus mitigating the inhibitory effect of miRNA on its target genes [22, 23]. For example, circ_0032131 promoted OA progression by sponging miR-502-5p and regulating the protein level of PRDX3 [24]. Circ_DHRS3 sponged miR-138-5p to elevate GREM1 expression in chondrocytes [25]. MiR-186-5p was increased in IL-1β-induced chondrocytes, and its overexpression suppressed OA progression by regulating the expression of MAPK1 [26]. CircHIPK3 knockdown inhibited osteoarthritis chondrocyte apoptosis by up-regulating miR-124 and down-regulating SOX8 [23]. Moreover, miR-338-3p was up-regulated in an OA model [27]. SRY-related high mobility group-box gene 9 (SOX9) has been reported to inhibit the progression of OA [28]. However, the relationships among circ_0008365, miR-338-3p, and SOX9 have not been reported. In this study, we explored the roles of circ_0008365 in OA progression. Moreover, we analyzed the circ_0008365/miR-338-3p/SOX9 regulatory pathway in the occurrence of OA in vitro with the hope of providing a possible therapeutic target for OA patients.

## Materials and methods

### Tissue sample

OA cartilage tissues (*n* = 25) were isolated from OA patients, and normal cartilage tissues were collected from knee joints of other patients without OA. All patients were recruited from Tianjin Hospital. This study was approved by the Ethics Committee of Tianjin Hospital. All patients provided the written informed consents.

### Cell culture and transient transfection

Human chondrocyte cell line (CHON-001) and 293 T cells were obtained from ATCC (Manassas, VA, USA). All cells were incubated in Roswell Park Memorial Institute-1640 (RPMI-1640; Gibco, Carlsbad, CA, USA) medium supplemented with 10% fetal bovine serum (FBS; Gibco). To mimic an in vitro cell model for OA, CHON-001 cells were induced by IL-1β (10 ng/mL, Sigma Aldrich, St. Louis, MO, USA) for 24 h as instructed [29]. pcDNA-circ_0008365 (the overexpression vector of circ_0008365), miR-338-3p mimic or inhibitor (miR-338-3p or anti-miR-338-3p), siRNA against SOX9 (si-SOX9) or corresponding controls (pcDNA, miR-NC, anti-NC, and si-NC) were purchased from RiboBio (Guangzhou, China) and transfected into cells by using Lipofectamine™ 3000 kit (Invitrogen), followed by transfection for 24 h for further studies.

### Quantitative real-time polymerase chain reaction (qRT-PCR)

TRIzol reagent (Invitrogen) was used to isolate total RNA. RNA was subjected to reverse transcription using a PrimeScript RT reagent kit (Takara, Tokyo, Japan). Then, a SYBR Premix Ex Taq II kit (Takara) was used to perform qPCR-PCR. The 2^−∆∆Ct^ method was used for quantification analysis. Sequences are listed in Table 1.

**Table 1:** Primers sequences used for PCR

Name		Primers for PCR (5′–3′)
circ_0008365	Forward	AAGAAACGCACTTTCGTGGC
	Reverse	AAGGACGACCACACCGGAA
SOX9	Forward	AGGAAGTCGGTGAAGAACGG
	Reverse	CGCCTTGAAGATGGCGTTG
miR-338-3p	Forward	GTATGATCCAGCATCAGTGATT
	Reverse	CTCAACTGGTGTCGTGGAG
GAPDH	Forward	GACAGTCAGCCGCATCTTCT
	Reverse	GCGCCCAATACGACCAAATC
U6	Forward	CTCGCTTCGGCAGCACA
	Reverse	AACGCTTCACGAATTTGCGT
SERPINE2	Forward	ATGAGTGACTGCAGGTCGT
	Reverse	CCCGTGTTGGAGCCTAGTTC

### Cell Counting Kit-8 (CCK-8) assay

Transfected cells were plated into 96-well plates and incubated for 24 h. Then, CCK-8 reagent (Beyotime, Jiangsu, China) was added into the cells and cultured for 4 h. The optical density (OD) value at 450 nm was analyzed.

### 5-Ethynyl-2′-deoxyuridine (EDU) assay

Transfected cells were plated into 96-well plates and cultured for 48 h. An BeyoClick™ EdU-647 kit (Beyotime) was used for EDU assay. Cells were incubated with EDU buffer for 4 h. After that, 4% formaldehyde was used to fix the cells and cell nuclei were stained using DAPI. Lastly, the images were photographed.

### Western blot (WB) analysis

The proteins were isolated by RIPA buffer (Beyotime). The proteins were separated by sodium dodecyl sulfate–polyacrylamide gel electrophoresis (SDS–PAGE) and transferred to polyvinylidene difluoride (PVDF) membranes (Millipore, Billerica, MA, USA). These membranes were then blocked with 5% non-fat milk (Beyotime) and incubated with primary antibodies overnight at 4℃.The primary antibodies, including anti-SOX9 (1:1000, ab185966, Abcam), anti-PCNA (1:1000, 13110S, CST), anti-cleaved caspase-3 (1:1000, 9661S, CST), anti-MMP13 (1:1000, 69926S, CST), anti-ADAMTS5 (1:1000, ab41037, Abcam), anti-COL2A1 (1:1000, ab188570, Abcam), anti-Aggrecan (1:1000, ab3778, Abcam), or anti-β-actin (1:2,000, ab8227, Abcam). After secondary antibodies were incubated with the membranes, protein bands were developed with an ECL kit (Solarbio, Beijing, China).

### Dual-luciferase reporter assay

The fragments of circ_0008365 and 3′UTR of SOX9 containing miR-338-3p-binding sites and the corresponding mutated sequences were synthesized and individually cloned into the psiCHECK2 vector (Promega, Madison, WI, USA), generating wild-type plasmids (circ_0008365-WT and SOX9-3′UTR-WT) and mutant-type plasmids (circ_0008365-MUT and SOX9-3′UTR-MUT). Cells were co-transfected with reporter plasmids and miR-338-3p mimic or miR-NC using Lipofectamine™ 3000 reagent. After that, luciferase activities were detected using Dual Luciferase Reporter Gene Assay Kit (Yeasen, Shanghai, China).

### RNA pull-down assay

Biotinylated-miR-con (bio-miR-con) and bio-miR-338-3p wild-type (bio-miR-338-3p) were obtained from RiboBio and transfected into cells for 48 h. After that, the cells were lysed by RIPA buffer (Beyotime), followed by incubation with magnetic beads After washing the beads, circ_0008365 enrichment in the RNA complexes was analyzed by qRT-PCR.

### RNA Immunoprecipitation (RIP) assay

Magna RIP Kit (Abcam, Cambridge, UK) was used for RIP assay. After cells were lysed, cell lysates were co-cultured with Ago2 antibody-bound beads or IgG antibody-bound beads overnight at 4 °C. RNA level was assessed by qRT-PCR.

### Flow cytometry analysis

Annexin V-FITC Apoptosis Detection Kit (Beyotime) was used to analyze cell apoptosis. Briefly, cells were collected and resuspended in 1 × binding buffer. Next, Annexin V-FITC and PI were used to incubate these cells. After that, flow cytometry (Becton, USA) was used to analyze cell apoptosis.

### Exosome isolation and identification

Collected plasma was centrifuged at 3000 g for 15 min to discard cell fragmentation. Exoquick exosome precipitation solution (System Biosciences) was used to isolate exosomes. Exosomes were observed by transmission electron microscopy (TEM). Exosome marker proteins (CD63 and TSG101) were identified by western blot.

### Statistical analysis

All experiments were repeated at least three times. Graphpad Prism 7.0 software was used to analyze data, and all data were presented as mean ± standard deviations. Differences between two groups were analyzed by Student’s *t* test, and differences among three or more groups were analyzed by one-way analysis of variance. *P* < 0.05 was considered statistically significant.

## Results

### Circ_0008365 expression was decreased in OA cartilage tissues and IL-1β-induced CHON-001 cells

**Fig. 1:**
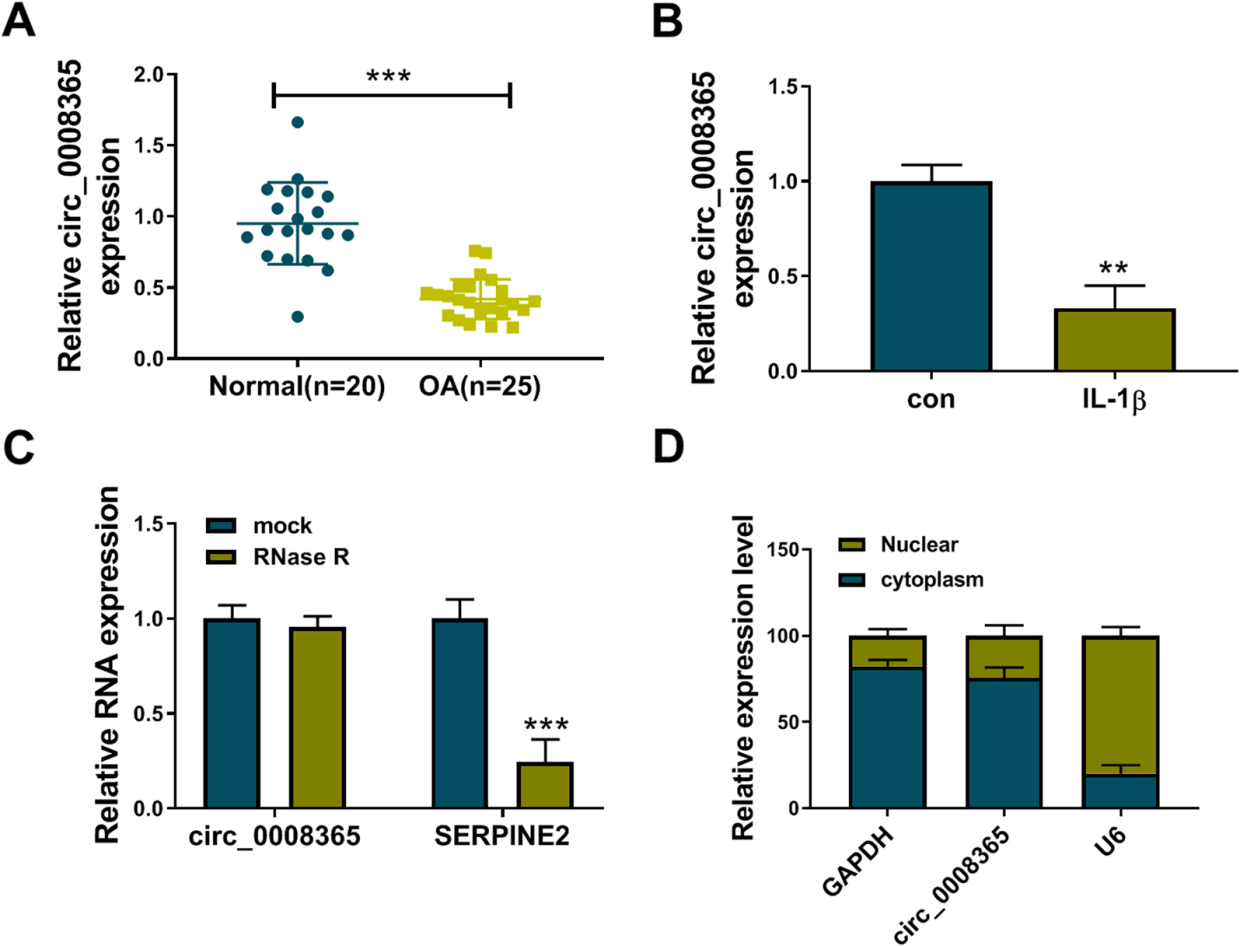
Circ_0008365 expression was decreased in OA cartilage tissues and IL-1β-induced CHON-001 cells. **A** Relative expression of circ_0008365 was detected in OA cartilage tissues (*n* = 25) and normal tissues (*n* = 20); **B** Relative expression of circ_0008365 was detected in IL-1β-induced CHON-001 cells; **C** The stability of circ_0008365 and SERPINE2 was analyzed by qRT-PCR in CHON-001 cells treated with or without RNase R; **D** The location of circ_0008365 was determined by cytoplasmic and nuclear RNA separation assay. **P < 0.01, ***P < 0.001

To explore the potential role of circ_0008365 in OA, we first detected the expression of circ_0008365 in OA cartilage tissues (*n* = 25) and normal tissues (*n* = 20). The results showed that circ_0008365 was decreased in OA cartilage tissues compared with normal tissues (Fig. 1A). Next, IL-1β-induced CHON-001 cells were used to mimic the OA in vitro cell model, and the results of qRT-PCR showed that circ_0008365 expression was also decreased in IL-1β-induced CHON-001 cells compared to untreated cells (Fig. 1B). The stability of circ_0008365 was assessed using RNase R, convergent primers, and divergent primers. RNase R had no effect on the expression of circ_0008365 but significantly impaired the expression of SERPINE2 mRNA (Fig. 1C). Circ_0008365 could only be amplified in cDNA by divergent primers (Additional file 1: Fig. S1). Then, cytoplasmic and nuclear RNA analysis showed that circ_0008365 was mainly located in the cytoplasm of CHON-001 cells (Fig. 1D).

### Circ_0008365 inhibited IL-1β-induced CHON-001 cell damage

**Fig. 2:**
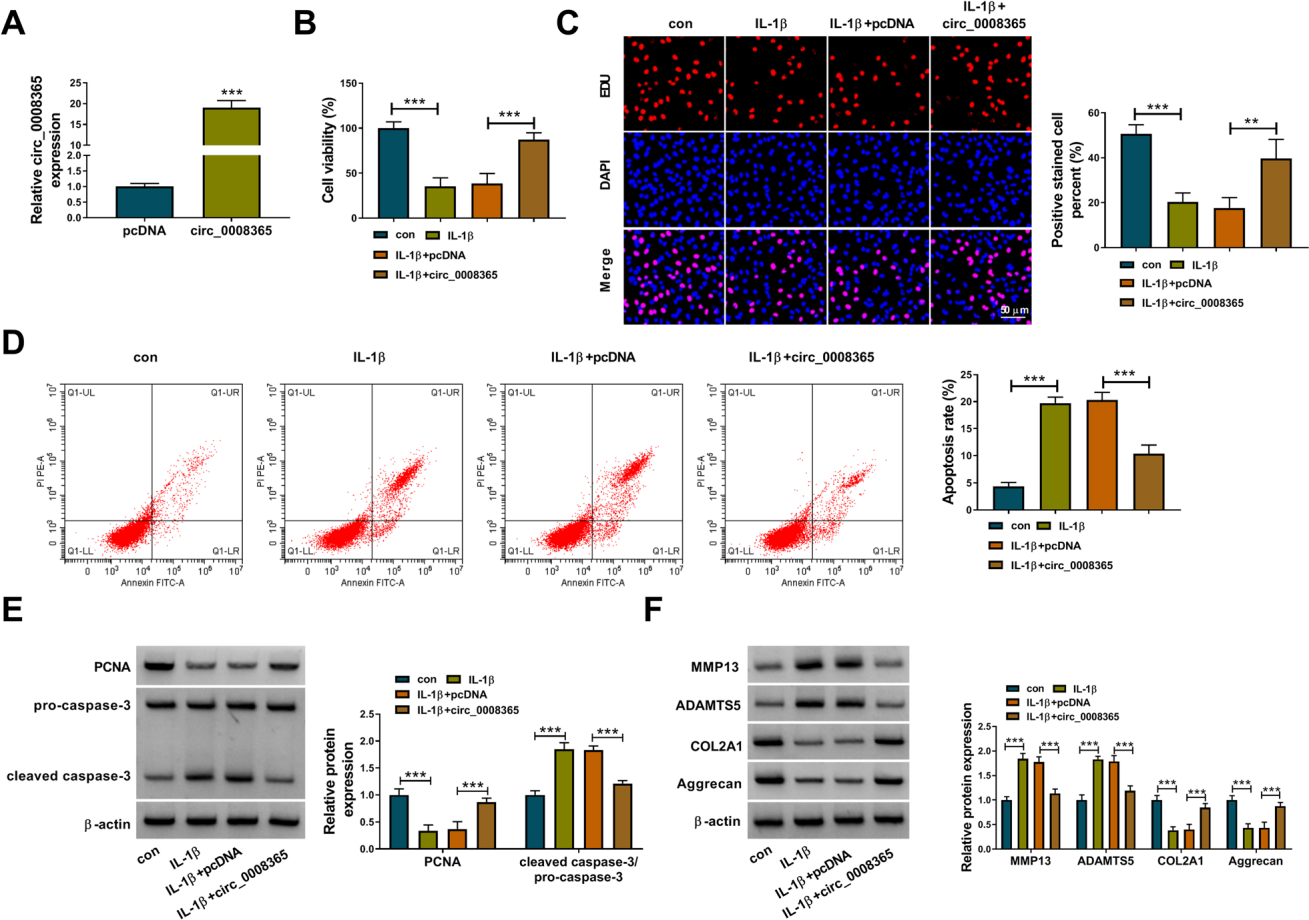
Circ_0008365 overexpression inhibited IL-1β-induced CHON-001 cell damage. **A** The expression of circ_0008365 was detected by qRT-PCR in CHON-001 cells transfected with pcDNA or circ_0008356; **B**–**F** CHON-001 cells were divided into 4 groups, including con, IL-1β, IL-1β + pcDNA, and IL-1β + circ_0008356 groups; (B) CCK-8 assay was used to detect cell viability; **C** EDU assay was used to measure cell proliferation; **D** Flow cytometry analysis was performed to evaluate the apoptosis of cells; **E**, **F** Western blot assay was employed to detect the protein levels of proliferation-related PCNA, apoptosis-related pro-caspase-3 and cleaved caspase-3 and ECM-related protein (MMP13, ADAMTS5, COL2A1, and Aggrecan). **P < 0.01, ***P < 0.001

To determine the function of circ_0008365 in IL-1β-induced CHON-001 cell damage, we overexpressed circ_0008365 in CHON-001 cells (Fig. 2A). CHON-001 cells were transfected with pcDNA or pcDNA-circ_0008365 (circ_000835) and then treated with IL-1β for 24 h. As shown in Fig. 2B, C, the results of CCK-8 assay and EDU assay demonstrated that overexpression of circ_0008365 rescued the inhibitory effect of IL-1β on CHON-001 cell viability and proliferation. Flow cytometry analysis showed that overexpression of circ_0008365 partially rescued cell apoptosis induced by IL-1β (Fig. 2D). In addition, the expression of PCNA was decreased and cleaved caspase-3 expression was increased in IL-1β-treated CHON-001 cells, while overexpression of circ_0008365 partially increased the protein level of PCNA and inhibited the activation of caspase-3 in IL-1β-treated CHON-001 cells (Fig. 2E). Furthermore, western blot assay showed that IL-1β treatment increased MMP13 and ADAMTS5 levels and decreased COL2A1 and Aggrecan levels in CHON-001 cells, while circ_0008365 overexpression reversed these impacts (Fig. 2F). Taken together, overexpression of circ_0008365 promoted proliferation and suppressed apoptosis and ECM degradation of IL-1β-treated CHON-001 cells.

### Circ_0008365 functioned as a sponge of miR-338-3p

**Fig. 3:**
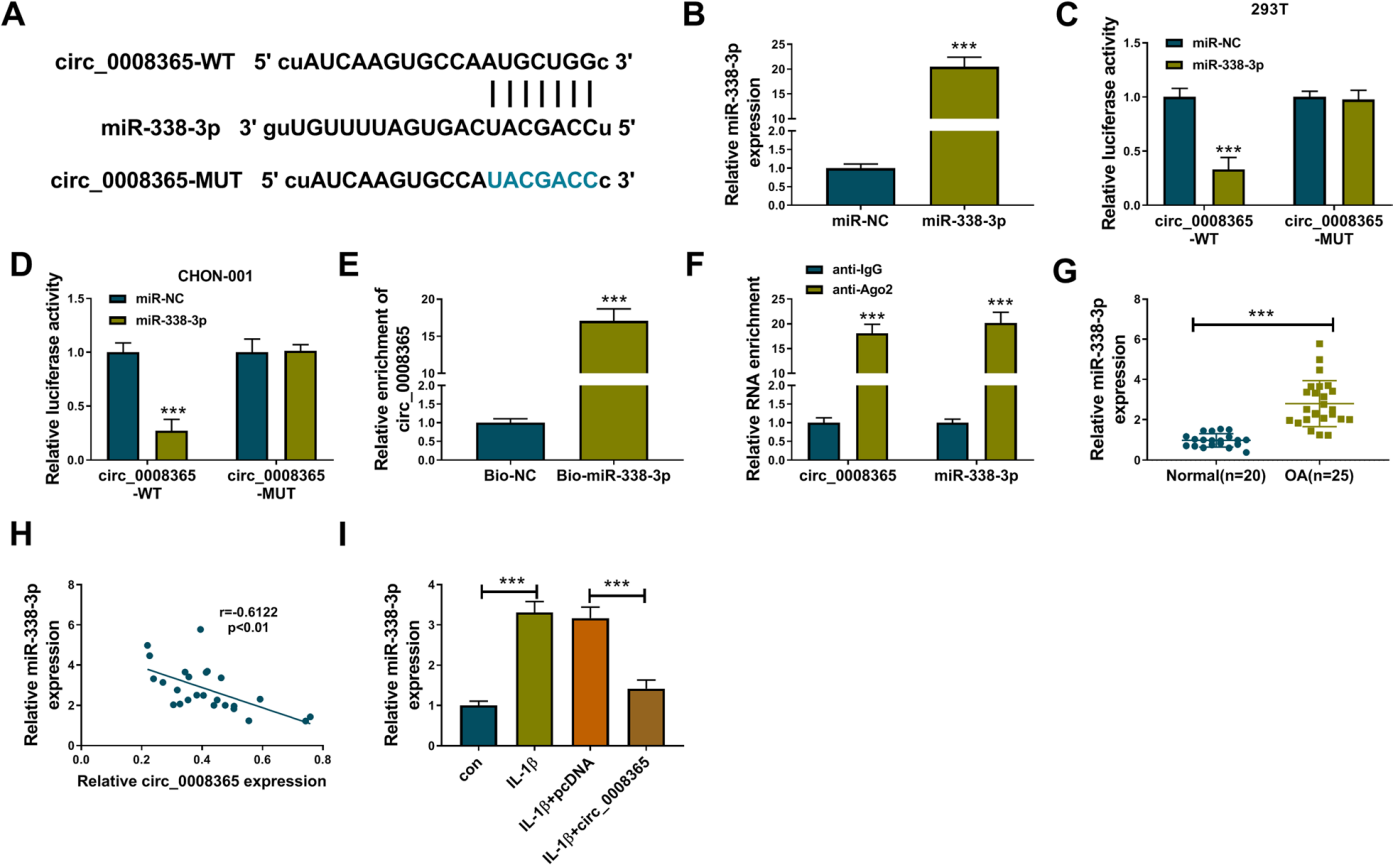
MiR-338-3p was a target of circ_0008365. **A** The binding sequence between circ_0008365 and miR-338-3p was predicted by starbase online database; **B** The expression level of miR-338-3p was detected by qRT-PCR in CHON-001 cells transfected with miR-NC or miR-338-3p; **C**, **D** The luciferase activity in 293 T and CHON-001 cells co-transfected with miR-NC/miR-338-3p and WT-circ_0008365/MUT-circ_0008365 was detected by dual-luciferase reporter assay; **E** The enrichment of circ_0008365 in CHON-001 cells incubated with Bio-NC or Bio-miR-338-3p was detected by RNA pull-down assay; **F** The enrichments of circ_0008365 and miR-338-3p were measured by RIP assay; **G** The miR-338-3p expression in OA cartilage tissues (*n* = 25) and normal tissues (*n* = 20) was measured by qRT-PCR; **H** Pearson correlation analysis was carried out to reveal the relationship between miR-338-3p and circ_0008365 expression in OA tissues; **I** The expression of circ_0008365 in CHON-001 cells treated with IL-1β, IL-1β + pcDNA or IL-1β + circ_0008365 and control cells was detected by qRT-PCR assay. ***P < 0.001

Since circ_0008365 is distributed in the cytoplasm, we used the starbase online database to predict the potential targets of circ_0008365. As shown in Fig. 3A, miR-338-3p was a target of circ_0008365. Next, dual-luciferase reporter assay, pull-down assay, and RIP assay were used to verify the association between miR-338-3p and circ_0008365. The transfection efficiency of miR-338-3p mimic was proved by qRT-PCR (Fig. 3B). The relative luciferase activity of WT-circ_0008365 was suppressed by miR-338-3p mimic, while the relative luciferase activity of the MUT-circ_0008365 group was not changed by miR-338-3p introduction in 293 T and CHON-001 cells (Fig. 3C, D). Moreover, the results of RNA pull-down assay indicated that circ_0008365 could be pulled down by miR-338-3p probe (Fig. 3E). Meanwhile, Ago2 RIP experiments confirmed that both circ_0008365 and miR-338-3p could bind to Ago2 protein (Fig. 3F). These data demonstrated that miR-338-3p was a target of circ_0008365. In addition, miR-338-3p expression was up-regulated in OA cartilage tissues compared with normal tissues (Fig. 3G), and we also found a negative relationship between circ_0008365 and miR-338-3p expression in OA tissues (Fig. 3H). The expression of miR-338-3p was increased by IL-1β treatment, but overexpression of circ_0008365 inhibited the miR-338-3p expression (Fig. 3I). Taken together, our results indicated that circ_0008365 was associated with miR-338-3p.

### Circ_0008365 alleviated IL-1β-induced CHON-001 cell damage by binding to miR-338-3p

**Fig. 4:**
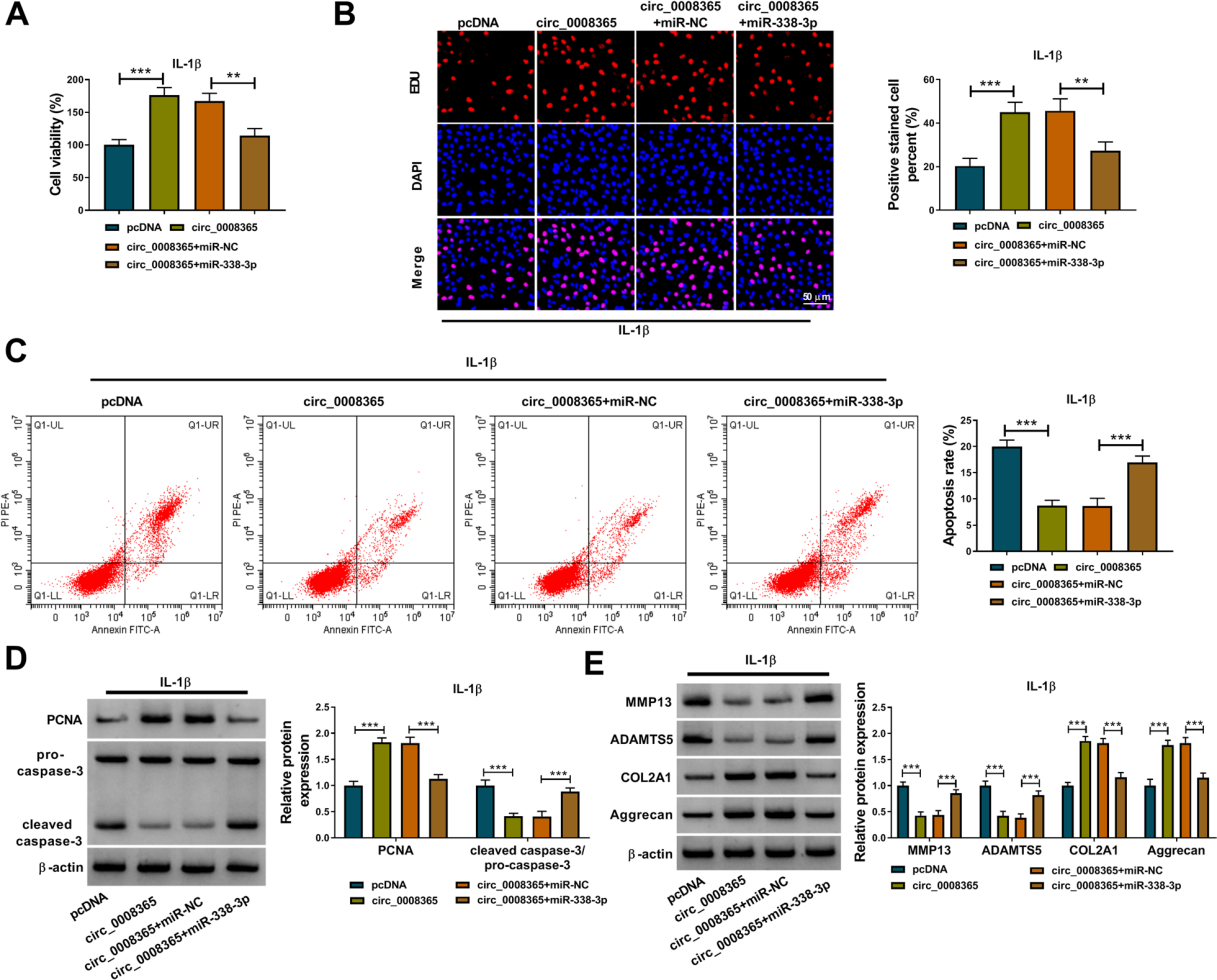
Circ_0008365 alleviated IL-1β-induced CHON-001 cell damage by binding to miR-338-3p. **A**–**E** IL-1β-induced CHON-001 cells were transfected with pcDNA, circ_0008365, circ_0008365 + miR-NC or circ_0008365 + miR-338-3p; **A** The viability of cells was examined by CCK-8; **B** EDU assay was conducted to detect cell proliferation; **C** The apoptosis of cells was measured by Flow cytometry analysis; **D**, **E** Western blot analysis was used to determine protein levels. **P < 0.01, ***P < 0.001

To study whether circ_0008365 regulated IL-1β-induced CHON-001 cell damage by binding to miR-338-3p, IL-1β-induced CHON-001 cells were transfected with pcDNA, pcDNA-circ_0008365 (circ_0008365), circ_0008365 + miR-NC or circ_0008365 + miR-338-3p. Cell viability and proliferation were promoted by circ_0008365 overexpression, but these effects were largely overturned by miR-338-3p overexpression in IL-1β-induced CHON-001 cells (Fig. 4A, B). Flow cytometry analysis showed that overexpression of circ_0008365 significantly decreased cell apoptotic rate in IL-1β-induced CHON-001 cells, which was reversed by up-regulating miR-338-3p (Fig. 4C). Meanwhile, the expression level of PCNA was elevated and cleaved caspase-3 was inhibited by circ_0008365 overexpression, but these effects were rescued by increasing miR-338-3p expression in IL-1β-induced CHON-001 cells (Fig. 4D). In addition, the data showed that overexpression of circ_0008365 restrained MMP13 and ADAMTS5 levels and enhanced COL2A1 and Aggrecan levels, while miR-338-3p reintroduction recovered MMP13 and ADAMTS5 levels and decreased COL2A1 and Aggrecan levels (Fig. 4E). The above findings implied that circ_0008365 regulated IL-1β-induced CHON-001 cell damage by binding to miR-338-3p.

### SOX9 was a direct target of miR-338-3p

**Fig. 5:**
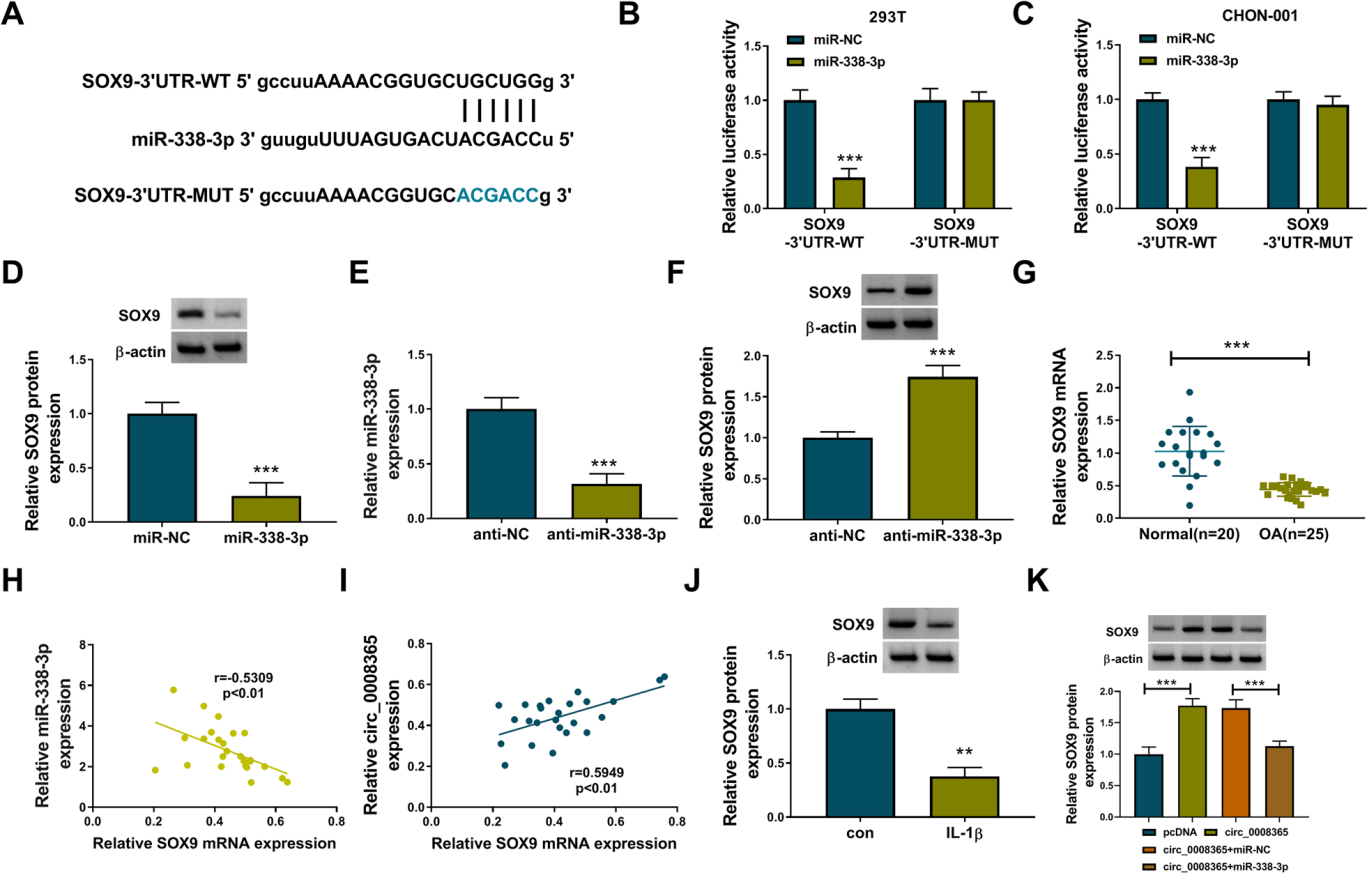
SOX9 was a direct target of miR-338-3p. **A** Starbase online database was used to predict the binding sites between miR-338-3p and SOX9; **B**, **C** Relative luciferase activities were detected in 293 T and CHON-001 cells co-transfected with miR-NC or miR-338-3p and SOX9-3′UTR-WT or SOX9-3′UTR-MUT; **D** Western blot assays were used to examine the protein level of SOX9 in CHON-001 cells after miR-338-3p overexpression; **E** The knockdown efficiency of miR-338-3p inhibitor was detected by qRT-PCR; **F** The protein level of SOX9 was detected in CHON-001 cells after miR-338-3p knockdown; **G** The relative level of SOX9 was detected in OA cartilage tissues (*n* = 25) and normal tissues (*n* = 20) by qRT-PCR; **H**, **I** The correlation between SOX9 and miR-338-3p or circ_0008365 expression level in OA tissues was analyzed by Pearson correlation analysis; (J) The protein level of SOX9 was assessed in IL-1β-induced CHON-001 cells; (K) The protein level of SOX9 was measured by western blot in IL-1β-treated CHON-001 cells transfected with pcDNA, circ_0008365, circ_0008365 + miR-NC or circ_0008365 + miR-338-3p. **P < 0.01, ***P < 0.001

SOX9 was predicted to be a potential downstream target gene of miR-338-3p by Starbase (Fig. 5A). Luciferase reporter assays showed that the luciferase activity was significantly reduced after the co-transfection of WT-3′UTR SOX9 with miR-338-3p mimics in 293 T and CHON-001 cells (Fig. 5B). Western blot analysis suggested that SOX9 expression was negatively regulated by miR-338-3p (Fig. 5D–F). Furthermore, the expression of SOX9 in OA tissues was down-regulated compared with normal tissues (Fig. 5G). Pearson correlation analysis showed that SOX9 expression was negatively correlated with miR-338-3p expression and positively correlated with circ_0008365 expression in OA tissues (Fig. 5H, I). Additionally, western blot results showed that the protein level of SOX9 was decreased in IL-1β-induced CHON-001 cells (Fig. 5J). Besides, the protein level of SOX9 was increased by the overexpression of circ_0008365 but could be restored by miR-338-3p (Fig. 5K). The above evidence suggested that miR-338-3p interacted with SOX9 and that circ_0008365 could regulate SOX9 expression by sponging miR-338-3p in CHON-001 cells.

### Knockdown of miR-338-3p inhibited IL-1β-induced CHON-001 cell damage by regulating SOX9 expression

**Fig. 6:**
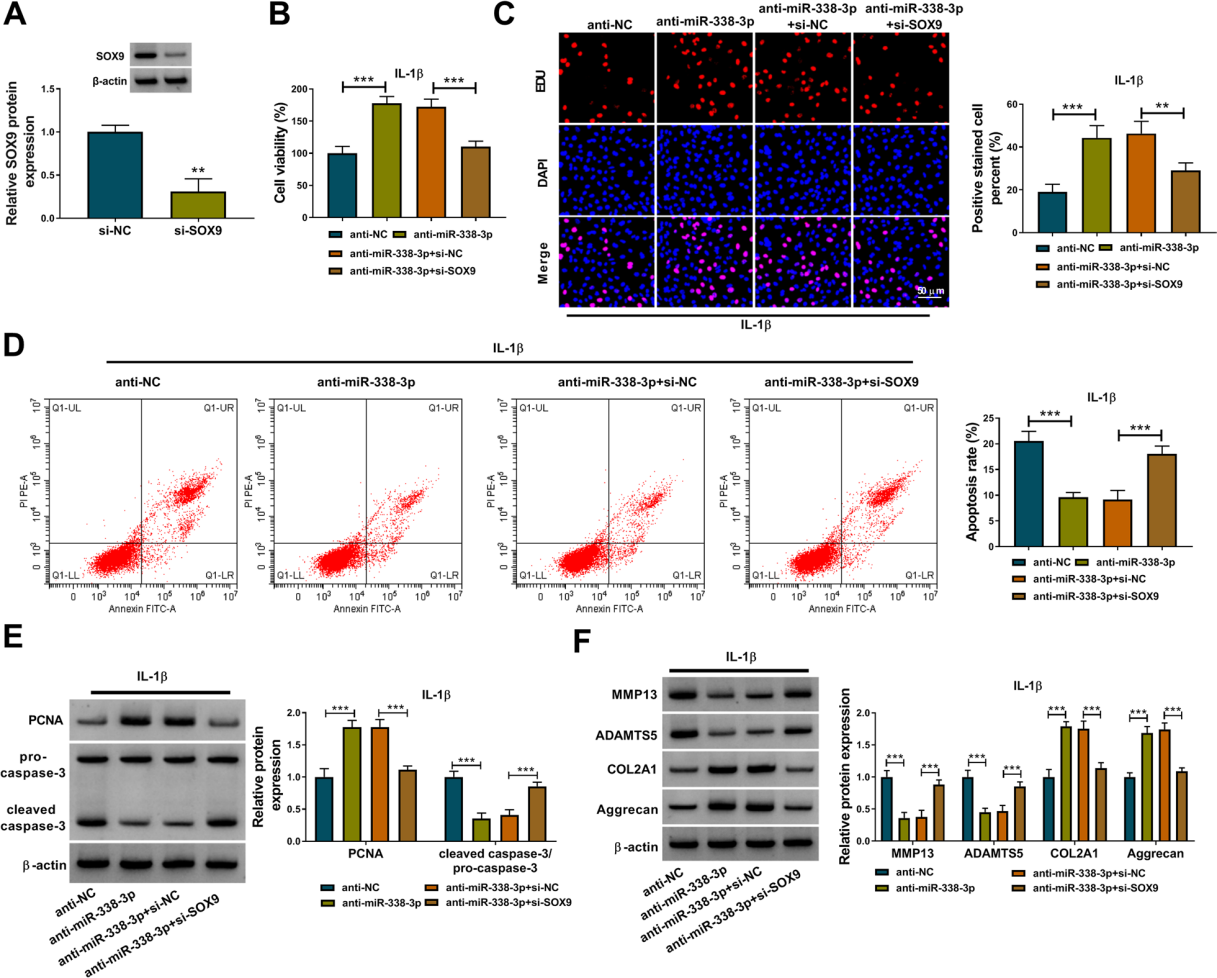
Knockdown of miR-338-3p inhibited IL-1β-induced CHON-001 cell damage by regulating SOX9 expression. **A** The transfection efficiency of SOX9 overexpression vector was assessed by western blot analysis; **B**–**F** IL-1β-treated CHON-001 cells were transfected with anti-NC, anti-miR-338-3p, anti-miR-338-3p + si-NC or anti-miR-338-3p + si-SOX9; CCK-8 assay (**B**), EDU assay (**C**), and flow cytometry analysis assay (**D**) were used to examine cell viability, proliferation, and apoptosis of cells; **E**, **F** The protein levels of proliferation-related PCNA, apoptosis-related pro-caspase-3 and cleaved caspase-3 and ECM-related protein (MMP13, ADAMTS5, COL2A1, and Aggrecan) were detected by western blot analysis. **P < 0.01, ***P < 0.001

To explore the association between miR-338-3p and SOX9 in IL-1β-induced CHON-001 cells, rescue assays were performed. The knockdown efficiency of SOX9 was proved by qRT-PCR and the result is shown in Fig. 6A. IL-1β-induced CHON-001 cells were transfected with anti-NC, anti-miR-338-3p, anti-miR-338-3p + si-NC or anti-miR-338-3p + si-SOX9. CCK-8 and EUD assays revealed that the knockdown of miR-338-3p significantly promoted cell viability and proliferation, but these effects were rescued by SOX9 knockdown in IL-1β-induced CHON-001 cells (Fig. 6B, C). SOX9 knockdown reversed miR-338-3p silencing-induced cell apoptosis inhibition in IL-1β-induced CHON-001 cells (Fig. 6D). In addition, SOX9 silencing significantly reversed the PCNA promotion and the caspase-3 activity suppression induced by miR-338-3p silencing in IL-1β-induced CHON-001 cells (Fig. 6E). Furthermore, miR-338-3p inhibition decreased the protein levels of MMP13 and ADAMTS5 and increased the protein levels of COL2A1 and Aggrecan, while these effects were reversed by silencing SOX9 in IL-1β-induced CHON-001 cells (Fig. 6F). These results suggested that miR-338-3p inhibition attenuated IL-1β-induced CHON-001 cell damage by regulating SOX9.

### Circ_0008365 is secreted by exosomes in the serum of OA patients

**Fig. 7:**
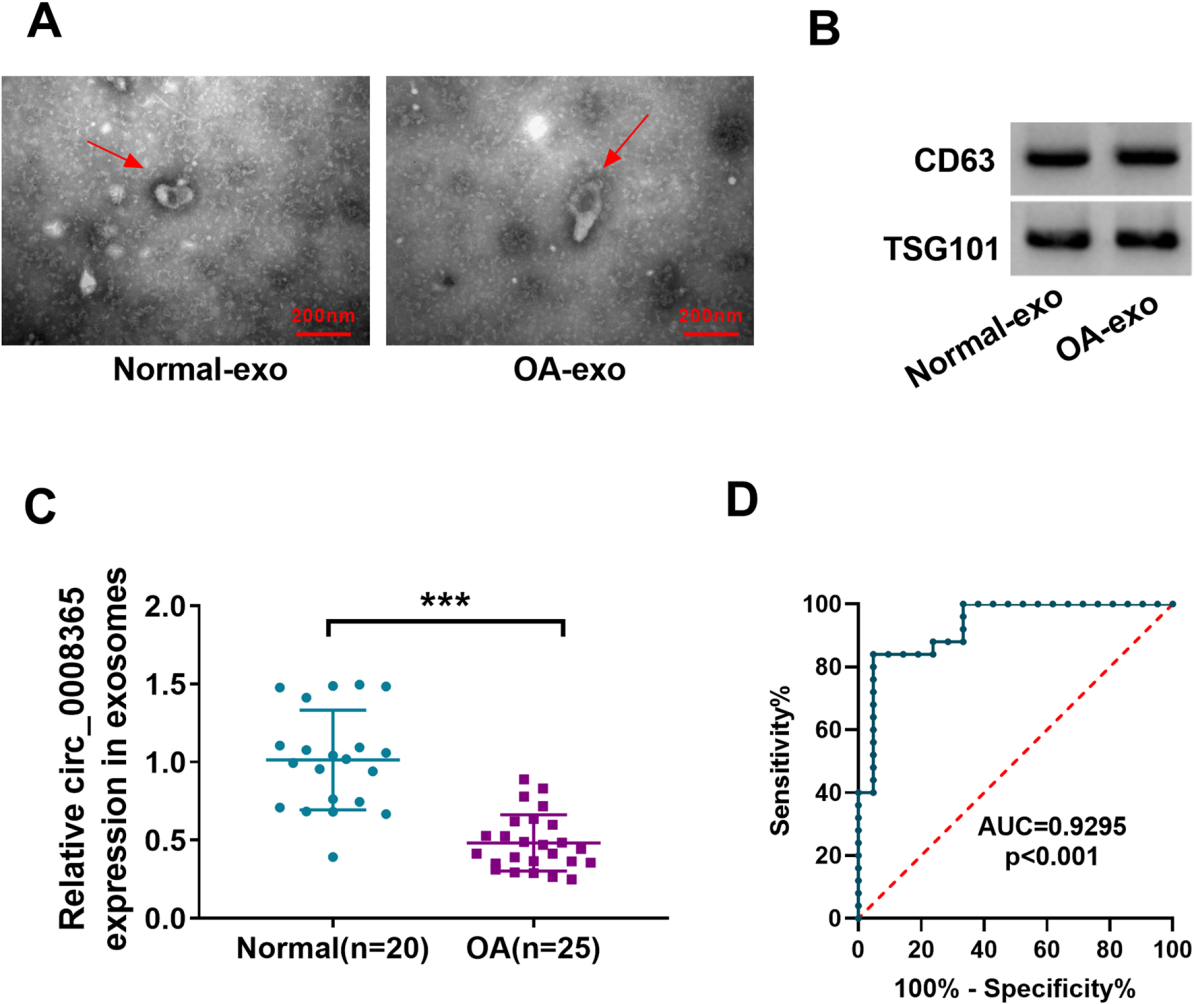
Circ_0008365 was secreted by exosomes in the serum of OA patients. **A** Exosomes (indicated by red arrows) derived from serum of OA patients and normal people were detected by electron microscope; **B** The protein levels of CD63 and TSG101 were measured by western blot assay; **C** The relative expression of circ_0008365 was measured by qRT-PCR in exosome derived from serum of OA patients (*n* = 25) and healthy controls (*n* = 20); **D** The receiver operating characteristic (ROC) curve analysis for exosomal circ_0008365 in diagnosing OA. ***P < 0.001

Finally, we collected serums from 25 OA patients and 20 normal subjects. After isolation of serum exosomes by sequential centrifugation, TEM analysis exhibited that normal-exo and OA-exo were round-shaped (Fig. 7A). The existence of exosome markers CD63 and TSG101 was confirmed by western blot (Fig. 7B). Then, we found that circ_0008365 expression is significantly higher in exosomes derived from the serum of OA patients compared with controls (Fig. 7C). Furthermore, ROC analysis results suggested that exosomal circ_0008365 had a diagnostic performance in OA with an AUC of 92.95% (Fig. 7D).

## Discussion

Recent research reports showed that circRNAs were involved in OA progression [30, 31]. For example, circ_0136474 expression was increased in OA patients and functioned as a sponge of miR-766-3p to elevate DNMT3A in OA chondrocytes [32]. CircRNA circSPG11 contributed to OA pathogenesis by negatively regulating miR-337-3p [33]. In our study, we found a new circRNA (circ_0008365) that played as an inhibitor in OA progression. We found that circ_0008365 and SOX9 were down-regulated and miR-338-5p was up-regulated in OA tissues and IL-1β-treated CHON-001 cells. Overexpression of circ_0008365 promoted proliferation and inhibited apoptosis and ECM degradation of IL-1β-induced CHON-001 cells. Exosomes are a class of extracellular vesicles with a size range of ~ 40 to 160 nm in diameter and contain diverse biomolecules, such as lipids, proteins, and nucleic acids [34]. Previous work has indicated the possibility of circRNAs as diagnostic markers [35]. Our data showed that exosomal circ_0008365 was down-regulated in the serum of OA patients in comparison with controls. Further, exosomal circ_0008365 had great value in diagnosing OA with an AUC of 0.9295.

MiRNAs play key roles in OA progression [36, 37]. For instance, MiR-145 was decreased in OA and could inhibit chondrocytes apoptosis by targeting BNIP3 and Notch signaling pathway [38]. MiR-410-3p expression was reduced in OA and LPS-induced chondrocytes and suppressed OA apoptosis and inflammation by sponging HMGB1, as revealed by an OA mouse model [39]. Li et al. found that miR-19b-3p and miR-17-5p were lowly expressed in OA and inhibited OA progression by negatively regulating EZH2 [40]. Overexpression of miR-675-3p constrained IL-1β-caused OA chondrocyte damage [41]. In this study, we found that circ_0008365 targeted miR-338-5p and that miR-338-5p could revert the effects of circ_0008365 on IL-1β-induced CHON-001 cell damage.

ECM degradation is a pivotal problem in OA, accompanied by aggrecan and collagen II down-regulation and ADAMTS5 and MMP13 up-regulation [42, 43]. SOX9 was involved in OA progression [44, 45]. SOX9 could regulate ADAMTSs-induced cartilage degeneration in human osteoarthritis [45]. In our study, we predicted that SOX9 was a downstream target gene of miR-338-5p and circ_0008365. Silencing of SOX9 overturned the effects of miR-338-5p inhibitor on IL-1β-induced damage.

Our studies also had some limitations. For example, inflammation is an important part of osteoarthritis. However, there is no detection of inflammation-related factors in our study. On the other hand, we only studied the role of circ_0008365 in OA using an in vitro cell model but not using in vivo mouse model. In future studies, we will focus on the above limitations.

In summary, overexpression of circ_0008365 inhibited IL-1β-induced CHON-001 cell damage by mediating the microR-338-5p/SOX9 pathway. Thus, the present work suggested that circ_0008365 might be used as a potential therapeutic target or diagnostic biomarker for OA.

## Supplementary Information

**Additional file 1: Fig. S1**. The circular structure of circ_0008365 was analyzed with divergent primers and convergent primers.

## Data Availability

Not applicable.
