# Supplementary figures and images for: Circular RNA circ_0008365 regulates SOX9 by targeting miR-338-3p to inhibit IL-1β-induced chondrocyte apoptosis and extracellular matrix degradation
Source: J Orthop Surg Res. 2022 Oct 14;17:452. doi: 10.1186/s13018-022-03240-z (PMC9569125; doi:10.1186/s13018-022-03240-z)

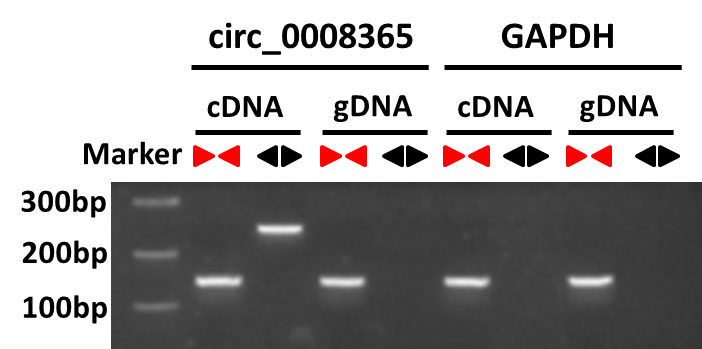

Supplement: Supplementary file 1 — Additional file 1: Fig. S1. The circular structure of circ_0008365 was analyzed with divergent primers and convergent primers. [file 13018_2022_3240_MOESM1_ESM.tif]
